# 非小细胞肺癌脑转移中肿瘤细胞与脑微环境互作机制研究进展

**DOI:** 10.3779/j.issn.1009-3419.2026.102.21

**Published:** 2026-07-20

**Authors:** Heqi YANG, Qinghua ZHOU

**Affiliations:** 610041 成都，四川大学华西医院肺癌研究所/肺癌中心; Lung Cancer Institute/Lung Cancer Center, West China Hospital, Sichuan University, Chengdu 610041, China

**Keywords:** 肺肿瘤, 脑转移, 肿瘤微环境, 单细胞测序, 空间组学, Lung neoplasms, Brain metastasis, Tumor microenvironment, Single-cell sequencing, Spatial omics

## Abstract

非小细胞肺癌（non-small cell lung cancer, NSCLC）脑转移的形成依赖肿瘤细胞与脑特异性微环境的持续互作。脑血管、胶质细胞及免疫细胞共同参与脑内定植、免疫逃逸和治疗抵抗；单细胞与空间组学进一步揭示了细胞状态、互作网络和空间生态位的异质性。本文综述NSCLC脑转移的发生与定植过程、脑内微环境及其互作机制，并讨论单细胞与空间组学揭示的新型异质性及其在治疗反应和患者分层中的潜在价值，为理解脑转移生态系统及微环境导向的精准治疗提供参考。

## Abstract (en)

Brain metastasis in non-small cell lung cancer (NSCLC) is shaped by continuous interactions between tumor cells and the brain-specific microenvironment. Vascular, glial and immune components contribute to intracranial colonization, immune escape and therapeutic resistance, while single-cell and spatial omics reveal heterogeneity in cellular states, communication networks and spatial niches. This review summarizes NSCLC brain metastasis and intracranial colonization, the brain metastatic microenvironment and tumor-microenvironment interactions, and discusses the heterogeneity revealed by single-cell and spatial omics and its potential relevance to therapeutic response and patient stratification.

肺癌是全球疾病负担最重的恶性肿瘤之一。2022年全球约有250万例新发肺癌和180万例死亡病例^[1]^。非小细胞肺癌（non-small cell lung cancer, NSCLC）约占肺癌的85%，主要包括肺腺癌（lung adenocarcinoma, LUAD）、鳞状细胞癌等亚型。尽管筛查、靶向治疗和免疫治疗持续改善NSCLC患者生存，远处转移仍是影响长期预后的关键因素^[2]^。脑转移发生于25%-50%的患者^[3]^，随着驱动基因阳性患者生存期延长以及全身治疗控制能力提高，脑转移及其相关治疗失败问题愈发突出^[4,5]^。

NSCLC脑转移并非肿瘤细胞播散至脑组织后的被动生长，而是一个涉及器官选择、屏障跨越、肿瘤微环境（tumor microenvironment, TME）适应和生态位重塑的动态过程^[6,7]^。肿瘤细胞从肺原发灶脱离后，需要经历循环存活、脑微血管滞留、血脑屏障（blood-brain barrier, BBB）跨越以及脑实质内存活和定植等连续环节。与多数外周器官不同，脑组织具有高度特化的血管屏障、神经胶质细胞网络、相对独特的免疫调控状态及代谢环境，这些特征既构成肿瘤细胞进入和适应脑组织的选择压力，也参与塑造支持转移细胞持续存活和扩增的器官特异性生态位。脑血管内皮细胞、周细胞、星形胶质细胞、小胶质细胞、外周来源髓系细胞、淋巴细胞及细胞外基质（extracellular matrix, ECM）等共同构成脑转移TME，并与肿瘤细胞形成持续、双向的相互作用^[8,9]^。因此，NSCLC脑转移更应被理解为肿瘤细胞与脑特异性TME共同演化的结果，而非单一肿瘤细胞内在改变或单条信号通路所能解释。

随着组学技术的发展，单细胞测序、空间组学及多重成像技术正在将NSCLC脑转移研究由单一细胞或分子机制推进至对细胞状态、互作网络和空间生态位的整体解析^[6,10]^。本文据此梳理脑转移发生与定植过程，重点总结脑TME及其与肿瘤细胞的互作，并讨论其组学异质性、治疗反应和临床转化价值。

## 1 NSCLC脑转移的发生与脑内定植

肿瘤转移具备显著的器官靶向特性，经典“种子-土壤”学说认为，具有转移潜能的肿瘤细胞只有在与其生物学特征相适配的靶器官TME中才能完成存活、定植和持续扩增^[11]^。对于NSCLC而言，脑组织特有的血管屏障、神经胶质细胞组成、免疫调控状态及代谢环境既构成肿瘤细胞转移的选择压力，也为能够适应脑内环境的细胞提供特定生态位。因此，NSCLC脑转移并非单一阶段或单一分子事件，而是由原发灶转移潜能获得、循环播散、脑血管滞留与BBB跨越，以及脑实质内定植等环节共同构成的连续过程^[12]^。在这一过程中，肿瘤细胞自身状态变化与靶器官TME重塑相互交织，并共同决定脑转移能否最终建立。

### 1.1 脑转移前状态与生态位形成

NSCLC脑转移的发生首先要求肿瘤细胞获得脱离原发灶、进入循环并在远端存活的能力。上皮-间质转化（epithelial-mesenchymal transition, EMT）及其伴随的迁移和侵袭能力增强，有助于肿瘤细胞脱离原发灶并进入转移级联过程，但这些改变并不足以解释脑转移的器官选择性。近年来，来自原发LUAD和配对脑转移样本的研究^[13]^提示，与脑转移相关的肿瘤细胞特征可在临床可见的颅内转移形成之前被检测到。NSCLC脑转移灶具有更高的染色体不稳定性（chromosomal instability, CIN），并富集神经样转录程序；其中部分CIN^high^、神经样肿瘤细胞状态在原发灶中已经低频存在，而在脑转移灶中明显富集，提示转移过程中可能存在对特定肿瘤细胞状态的选择。进一步的原发肿瘤研究也支持这一观点：基于原发LUAD组织建立的DNA甲基化模型能够区分后续脑转移风险不同的患者，提示部分与脑转移相关的表观遗传特征在颅内转移形成前即可被检测到^[14]^；此外，对完全切除I-IIIA期LUAD患者的长期随访显示，原发肿瘤中的TP53突变与术后脑转移风险增加有关^[15]^。这些结果表明，脑转移并非完全由肿瘤细胞进入脑组织后获得的新表型驱动，而可能部分建立在原发肿瘤既有异质性的基础上。

脑转移的早期发生还可能受到远端脑TME预先改变的影响，即脑转移前生态位（pre-metastatic niche, PMN）的形成。PMN强调在播散肿瘤细胞完成脑内定植之前，原发肿瘤来源的循环信号已可改变远端组织状态，从而降低后续转移过程所面临的组织屏障。与其他肿瘤类型相比，NSCLC脑PMN的疾病特异性证据仍相对有限，但近年来已开始从单纯概念推测转向机制验证。肿瘤来源细胞外囊泡可作用于脑内宿主细胞：NSCLC患者循环细胞外囊泡中富集的LINC00482能够改变小胶质细胞功能，并在动物模型中促进有利于脑转移的TME形成^[16]^；NSCLC细胞来源外泌体也可诱导星形胶质细胞凋亡并改变其细胞因子分泌谱，但这一证据主要来自体外模型，其在人类脑转移前阶段的生理意义尚未得到直接验证^[17]^。近期的研究进一步将代谢异常与这一过程联系起来。脑转移相关LUAD中胆碱能代谢增强可通过影响星形胶质细胞稳态和BBB完整性促进肿瘤细胞外渗，而胆固醇升高则可同时增强肿瘤细胞转移能力、降低Claudin-5相关屏障稳定性并改变小胶质细胞功能状态^[18,19]^。因而，现有NSCLC证据支持PMN并非由单一细胞或单一分泌因子构成，而更可能表现为脑血管屏障完整性下降、胶质细胞稳态改变及局部免疫调节共同参与的组织状态转换。但是，NSCLC脑PMN在人类患者中的形成时序及因果作用仍缺乏直接证据。

### 1.2 血管内渗与血行播散

肿瘤细胞脱离原发灶后需穿越基底膜和血管内皮完成血管内渗。LUAD模型相关研究^[20]^提示，部分CD44^+^肿瘤细胞可形成周细胞样状态并通过GPR124相关信号增强跨内皮迁移和脑转移能力，提示特定肿瘤细胞状态与血管界面的互作可影响进入循环的效率。

进入血液循环后，循环肿瘤细胞（circulating tumor cells, CTCs）需要承受血流剪切力、失去基质依附以及免疫清除等多重压力，因此进入循环本身并不等同于获得转移能力。CTCs既可单独存在，也可形成由多个肿瘤细胞组成的同型细胞簇，或与免疫细胞等形成异型细胞簇。近期对晚期NSCLC患者外周血的分析证实，循环中存在具有不同免疫细胞组成的异型CTCs簇，部分肿瘤相关循环细胞群与晚期疾病、进展及转移风险有关^[21]^。肺癌模型进一步显示，细胞聚集可增强CTCs对循环应激和免疫清除的耐受，其中类固醇受体共激活因子（steroid receptor coactivator, Src）/纤连蛋白1（fibronectin 1, FN1）信号参与维持细胞间黏附和簇状结构，干扰该通路可降低CTCs簇的循环存活及转移能力^[22]^。在完成入血后，部分播散肿瘤细胞随血流进入脑循环，并在脑微血管内发生滞留和黏附，为后续跨越BBB及脑实质定植创造条件。

### 1.3 脑微血管滞留与BBB跨越

随血行播散进入脑循环的肿瘤细胞首先需要在脑微血管内完成滞留和内皮黏附，继而跨越BBB进入脑实质。BBB主要由脑微血管内皮细胞及其紧密连接、周细胞和星形胶质细胞足突等共同维持，是限制CTCs进入中枢神经系统的重要结构屏障。与一般毛细血管相比，脑内皮细胞间连接更为致密，因此肿瘤细胞在脑微血管中的稳定停留及与内皮细胞建立有效黏附，是后续跨内皮迁移的重要前提。

NSCLC细胞与脑血管内皮之间的黏附受到多种细胞黏附分子的调控。NSCLC脑转移灶中ALCAM表达增加，功能实验^[23]^显示ALCAM缺失可降低肿瘤细胞与脑内皮细胞的黏附，并减少实验性脑转移形成，支持其参与NSCLC细胞在脑血管界面的滞留与跨内皮迁移。此外，NSCLC细胞可通过影响脑内皮糖被和E-selectin暴露增强其与脑微血管内皮的黏附；CD15/CD15s与E-selectin相互作用在体外生理流动条件下也被证实能够促进NSCLC细胞与脑内皮结合^[24,25]^。这些结果表明，脑微血管滞留并非单纯的机械截留，而同时受到肿瘤细胞与脑内皮之间黏附相互作用的调控。

稳定黏附后，肿瘤细胞需要进一步突破内皮屏障。NSCLC相关研究^[26]^显示，这一过程通常伴随脑微血管内皮紧密连接和屏障通透性的改变。肿瘤来源lnc-MMP2-2可通过miR-1207-5p-EPB41L5轴破坏脑微血管内皮紧密连接并增加BBB通透性，其抑制可减少实验性NSCLC脑转移。有研究^[27]^进一步发现，NSCLC脑转移中升高的miR-522-3p可通过调控TNS1并改变ZO-1和occludin等紧密连接蛋白，增强肿瘤细胞跨越BBB的能力，提示紧密连接重塑可能是不同脑转移相关分子最终汇聚的共同效应之一。

### 1.4 脑内定植与早期适应

肿瘤细胞跨越BBB后并不意味着脑转移已经建立。进入脑实质的播散肿瘤细胞仍需在陌生的神经组织环境中维持存活，并逐步恢复增殖能力。活体成像研究^[28]^显示，肺癌细胞外渗后常与脑微血管保持紧密接触，随后形成微小转移灶；但相当一部分播散细胞仍会死亡或长期处于非增殖状态，提示脑内定植是转移级联中又一个重要的选择瓶颈。与原发肺组织相比，脑实质在细胞组成、营养物质可利用性及代谢环境等方面均存在显著差异，因此成功定植不仅依赖肿瘤细胞存活，还要求其获得适应脑内环境的表型和代谢状态。

有关LUAD脑转移的研究^[29]^发现，脑转移肿瘤细胞呈现明显的状态异质性，并提示间质-上皮转化（mesenchymal-epithelial transition, MET）相关程序可能参与转移部位的增殖和定植，其中ATF3被认为是调控这一过程的候选转录因子。与此同时，脑组织特有的营养环境可驱动肿瘤细胞发生代谢适应：NSCLC模型中，IGF2BP3通过稳定FASN mRNA增强脂质合成，从而提高肿瘤细胞在脑内的生长和定植能力^[30]^；另一LUAD研究^[31]^则显示，脑源性信号可诱导神经生长因子诱导蛋白（nerve growth factor inducible, VGF）表达，通过维持线粒体膜脂稳态和氧化磷酸化满足脑内生长所需的能量需求，抑制该过程可显著降低实验性脑定植。这些研究表明，脑内定植并非单纯延续原发灶的增殖程序，而伴随肿瘤细胞对脑组织环境的进一步适应。成功存活的肿瘤细胞随后与脑血管、星形胶质细胞、小胶质细胞及浸润性免疫细胞建立持续的双向互作，并逐渐形成能够支持肿瘤扩增的脑转移TME。

## 2 NSCLC脑转移TME及细胞互作

脑转移灶的建立并非肿瘤细胞进入脑实质后的自主扩增，而依赖其与局部脑TME的持续适应和相互塑造。脑血管相关细胞、星形胶质细胞、小胶质细胞及外周来源免疫细胞共同参与肿瘤细胞存活、增殖、免疫逃逸和治疗反应，并通过ECM和可溶性信号形成高度动态的局部TME。不同细胞成分的功能又具有明显的空间和状态依赖性，因此，NSCLC脑转移TME更适合从细胞状态及其相互作用的角度理解，而非将各类细胞视为彼此独立的促癌或抑癌成分。

### 2.1 脑血管及血管周微环境

脑血管在转移灶形成后仍参与肿瘤生长和治疗反应。NSCLC脑实质转移中可富集血管生成型内皮细胞，并伴随质膜囊泡相关蛋白（plasmalemma vesicle associated protein, PLVAP）、CD34及IV型胶原α1链（collagen type I α1, COL4A1）/COL4A2等血管和基底膜相关基因升高；血管丰富区域同时具有更高的肿瘤细胞增殖活性^[32]^。这些结果提示脑转移血管重塑同时涉及内皮状态和血管周ECM改变。

脑血管周区域还可能为转移细胞持续存活提供支持。表皮生长因子受体（epidermal growth factor receptor, EGFR）突变NSCLC模型显示，奥希替尼治疗后的残存肿瘤细胞可聚集于脑微血管周围，并通过血管共选择维持生存，RhoA相关信号参与这一过程及后续耐药性脑转移形成^[33]^。因此，脑血管不仅是肿瘤细胞进入脑实质的通道，也可成为支持肿瘤细胞残存和再生长的局部TME。

### 2.2 星形胶质细胞

星形胶质细胞是NSCLC脑转移TME中重要的脑固有细胞。肿瘤细胞进入脑实质后可诱导星形胶质细胞发生反应性改变，后者进一步通过直接细胞接触、旁分泌信号及代谢支持影响肿瘤细胞的存活和扩增。较早的肺癌脑转移模型已揭示一种具有代表性的双向通讯机制：脑转移细胞表达原钙黏蛋白7（protocadherin 7, PCDH7），促进其与星形胶质细胞形成由连接蛋白43（connexin 43, Cx43）组成的间隙连接，使环鸟苷酸-腺苷酸（cyclic GMP-AMP, cGAMP）由肿瘤细胞转移至星形胶质细胞并激活干扰素基因刺激因子（stimulator of interferon genes, STING）信号，继而诱导干扰素α（interferon-alpha, IFN-α）和肿瘤坏死因子（tumor necrosis factor, TNF）释放，反向激活肿瘤细胞信号传导及转录激活蛋白1（signal transducer and activator of transcription 1, STAT1）和核因子κB（nuclear factor kappa-B, NF-κB）通路，从而增强脑内生长及化疗抵抗^[34]^。这一机制提示，星形胶质细胞并非单纯提供结构支持，而可被脑转移细胞重塑为促进肿瘤存活的功能性伙伴。

近期NSCLC研究进一步拓展了这种肿瘤-星形胶质细胞互作的机制范围。脑TME中的星形胶质细胞可通过Wnt-5a相关信号诱导肺癌细胞代谢型谷氨酸受体1（metabotropic glutamate receptor 1, mGluR1）表达，并以谷氨酸依赖方式稳定EGFR、维持下游细胞外调节蛋白激酶（extracellular signal-regulated kinase, ERK）信号，从而形成脑内环境特异性的生存依赖^[35]^。在EGFR突变NSCLC脑转移中，反应性星形胶质细胞分泌的白细胞介素-11（interleukin-11, IL-11）还可激活肿瘤细胞EGFR及IL-11Rα/gp130相关信号，上调程序性细胞死亡配体1（programmmed cell death ligand 1, PD-L1）并促进CD8^+^ T细胞凋亡，将星形胶质细胞介导的肿瘤支持进一步延伸至局部免疫逃逸^[36]^。

亦有研究^[37]^提示，星形胶质细胞还可参与跨细胞网络的构建。肺癌脑转移细胞分泌的脂质运载蛋白2（lipocalin-2, LCN2）可结合星形胶质细胞SLC22A17并激活JAK2/STAT3信号，诱导星形胶质细胞活化和趋化因子分泌，进而募集巨噬细胞；后者释放IL-1β又可反向增强肿瘤细胞LCN2表达，形成肿瘤细胞-星形胶质细胞-巨噬细胞之间的正反馈环路。因此，现有NSCLC证据更支持将星形胶质细胞理解为可被肿瘤重塑并参与脑内适应、免疫调控和多细胞互作的动态TME成分，而非固定的促癌或抑癌细胞类型。

### 2.3 小胶质细胞与外周来源巨噬细胞

脑转移灶中的肿瘤相关髓系细胞主要由脑实质常驻小胶质细胞和外周单核细胞来源巨噬细胞（monocyte-derived macrophages, MDMs）共同构成。两者虽然共享CD68、IBA1等部分髓系标志物，但具有不同的发育来源：小胶质细胞主要来源于胚胎期卵黄囊祖细胞并可在脑内自我更新，而MDMs则由循环单核细胞募集后分化形成。更重要的是，当前单细胞和空间组学研究^[38]^已表明，小胶质细胞并不存在稳定且相互排斥的“M1/M2”表型，而是可根据局部环境刺激进入连续、动态的转录和功能状态。因此，在NSCLC脑转移中，将脑内髓系细胞简单归类为“M1型”或“M2型”已难以反映其真实异质性。

有关NSCLC临床样本的进一步研究^[39]^显示，这两类髓系细胞在脑转移灶中的组成并不相同。配对原发肺肿瘤和脑转移灶分析发现，脑转移灶中巨噬细胞显著增加；TMEM119免疫染色进一步提示，多数CD68^+^细胞缺乏小胶质细胞标志物，更符合外周来源巨噬细胞特征。空间转录组研究^[40]^则显示，NSCLC脑转移TME中小胶质细胞的TMEM119、P2RY12和CX3CR1等稳态标志降低，而吞噬和抗原呈递相关程序增强；与此同时，MDMs在部分高度纤维化区域明显富集，提示脑转移可同时重塑常驻小胶质细胞状态并促进外周髓系细胞进入。此外，脑转移区域还伴随COL1A1、COL3A1、FN1及基质金属蛋白酶2（matrix metallopeptidase 2, MMP2）等基质相关基因表达增加，提示髓系细胞重塑与局部纤维化及ECM改变可能在空间上相互耦联。

肿瘤来源信号可进一步决定这些髓系细胞的功能状态。NSCLC模型^[41]^显示，IL-6/JAK2/STAT3信号可诱导小胶质细胞形成以抗炎和促肿瘤功能为主的状态，并促进脑内定植；另一项研究^[42]^表明，NSCLC来源的巨噬细胞迁移抑制因子（macrophage migration inhibitory factor, MIF）可通过CD74调节小胶质细胞功能，阻断MIF-CD74轴能够增强放疗相关抗肿瘤效应。最近的研究^[43]^进一步显示，NSCLC脑转移灶中富集PLTP^+^肿瘤相关巨噬细胞（tumor-associated macrophages, TAMs）及特定单核细胞状态，支持脑转移髓系TME由多个功能不同的细胞群共同组成，而非单一“免疫抑制型巨噬细胞”群体。

总体而言，NSCLC脑转移中的髓系TME应被理解为脑驻留小胶质细胞状态转换与外周来源巨噬细胞募集共同形成的动态系统。目前两者的相对贡献、空间分工及随疾病进展和治疗变化的状态转换仍缺乏纵向研究，这也限制了对髓系细胞靶向策略的精准设计。

### 2.4 T细胞及免疫抑制TME

T细胞是NSCLC脑转移局部抗肿瘤免疫的主要效应细胞，但脑转移灶的免疫状态与原发肺肿瘤并不一致。配对原发灶和脑转移灶研究^[39]^显示，脑转移灶中T细胞相关免疫程序受到抑制，T细胞受体克隆丰富度降低，并伴随CD4^+^和CD8^+ ^T细胞浸润减少。另一项包含43例配对NSCLC样本的研究^[44]^同样发现，脑转移灶总体肿瘤浸润淋巴细胞减少、免疫相关特征评分下降，并更少呈现PD-L1高表达/CD8A高表达的炎症型免疫表型。这些结果共同提示，NSCLC脑转移并非简单延续原发灶的免疫状态，而是在脑内形成相对低淋巴细胞浸润的免疫环境。

这种免疫抑制并非仅表现为T细胞数量减少，还具有明显的肿瘤分子背景依赖性。LUAD脑转移研究^[45]^显示，EGFR/间变性淋巴瘤激酶（anaplastic lymphoma kinase, ALK）阳性脑转移总体具有更低的CD8^+^ T细胞和细胞毒性淋巴细胞浸润；其中EGFR阳性脑转移还表现出调节性T细胞（regulatory T cells, Tregs）相对增加，而较高的CD8A表达和细胞毒性淋巴细胞浸润与更好的生存结局有关。这提示脑转移免疫抑制并非单一模式，而可能受到驱动基因背景以及肿瘤细胞与局部TME共同调控。因此，NSCLC脑转移免疫TME的核心特征更适合概括为有效T细胞浸润不足与抗肿瘤功能受限并存。Tregs、髓系细胞、星形胶质细胞及免疫检查点信号均可能参与这一状态的维持，但这些机制并非彼此独立：前文所述星形胶质细胞和髓系细胞重塑最终均可汇聚于对效应T细胞功能的限制。

## 3 单细胞与空间组学揭示的NSCLC脑转移异质性

单细胞与空间组学研究表明，NSCLC脑转移并非由相对均一的肿瘤细胞和TME构成，而是由多种肿瘤细胞状态、免疫细胞亚群及空间生态位共同组成。对这些异质性的解析，为理解脑内适应、免疫重塑及潜在治疗靶点提供了新的依据。

### 3.1 细胞状态异质性

单细胞研究^[46]^显示，NSCLC脑转移恶性细胞具有明显的基因组和转录异质性。整合原发LUAD与脑转移数据^[47]^发现，脑转移恶性细胞具有较高拷贝数变异负荷并形成多个转录状态不同的细胞群；克隆和拟时序分析进一步识别出与脑转移相关的上皮状态，并提示S100A9^+^恶性细胞可能参与转移形成。结合既往多模态研究^[13,29]^，CIN、神经样程序及MET相关变化提示部分脑转移相关状态可能在原发灶中预存，并在转移过程中受到选择或重塑。

脑转移TME中的细胞同样表现出明显的状态异质性。单细胞和空间研究^[40,43]^显示，脑转移灶中的小胶质细胞逐渐丢失部分稳态特征，而外周来源巨噬细胞可形成多个转录状态不同的肿瘤相关亚群；T细胞内部也存在记忆、应激及功能受限等不同状态，其中近期研究^[43]^识别出脑转移中富集的HSP70^high ^T细胞和PLTP^+^ TAMs。因此，NSCLC脑转移TME的异质性不仅表现为不同细胞类型比例的变化，更体现在同一细胞谱系内部功能状态的重新分配。这一认识也为进一步分析不同细胞状态之间如何建立通讯网络提供了基础。

### 3.2 细胞互作网络

单细胞通讯分析提示，NSCLC脑转移过程中不仅发生细胞状态改变，不同细胞群之间的信号连接也随之重组。对原发肺癌及不同转移部位的比较显示，脑转移灶中肿瘤细胞与T细胞、B细胞及髓系细胞之间均出现区别于原发灶和淋巴结转移灶的配体-受体组合，提示脑组织环境可能形成具有器官部位特征的通讯网络^[48]^。进一步研究^[49]^发现，脑转移相关上皮细胞可通过MIF-CD74/CXCR4和MIF-CD74/CD44等信号与免疫细胞建立联系。这些结果将“肿瘤细胞状态改变”和“免疫细胞状态重组”连接起来，提示不同细胞状态并非彼此独立，而可能通过特定通讯轴形成相互依赖的功能网络。

空间多组学研究进一步支持这种网络重构。在具有脑转移倾向的NSCLC原发灶侵袭前沿，IGHG1^+^恶性上皮细胞与肌成纤维样癌相关成纤维细胞（myofibroblastic cancer-associated fibroblasts, myCAFs）呈显著空间共定位，并通过MIF-CD74和APP-CD74形成双向通讯；其中CD74相关互作还获得临床样本及体外实验验证^[50]^。综合现有证据，CD74相关信号可能是连接不同脑转移相关肿瘤和TME细胞状态的候选通讯节点，而非局限于单一细胞对之间的孤立通路。不过需要注意，现有多数配体-受体关系来源于转录表达推断，其方向性和功能作用仍需空间邻近、蛋白水平及干预实验进一步验证。

### 3.3 空间生态位组织

空间组学的结果表明，NSCLC脑转移TME并非不同细胞随机混合，而具有明确的区域化组织特征。既往研究^[40]^通过空间转录组学将脑转移组织区分为肿瘤核心、肿瘤免疫TME和邻近脑TME，发现不同区域具有明显不同的转录程序，其中免疫调节与纤维化改变共同构成脑转移周围的重要功能模块。这一结果首先提示，脑转移TME的异质性不仅来源于细胞组成差异，还表现为不同功能程序在组织空间中的区域化分布。

更高分辨率的空间多组学研究进一步揭示了这种区域化内部的细胞组织方式。对NSCLC脑转移灶进行多区域空间转录组和多重免疫组化分析，发现同一病灶内部可反复出现不同免疫细胞组合，其中髓系细胞和淋巴系细胞呈明显空间分隔，并受到不同细胞因子网络影响^[51]^。由此可见，单细胞分析预测出的通讯关系并非均匀发生于整个肿瘤，而可能集中于特定细胞邻域和肿瘤-免疫界面。

近期研究开始进一步从“空间分区”推进到具有明确功能的局部生态位。整合单细胞、空间转录组和代谢组学的NSCLC研究^[52]^发现，LOX^+^恶性细胞与特定中性粒细胞亚群在空间上共定位，后者形成中性粒细胞胞外诱捕网（neutrophil extracellular traps, NETs），并与局部脂质代谢重编程共同构成促脑转移生态位。多重免疫组化和单细胞空间统计研究^[53]^则显示，B7-H3^+^与B7-H3^-^ TAMs的局部混合和聚集程度与较差生存有关，而单纯计算细胞密度的预后价值相对有限。尽管后者仍属于小样本探索性研究，但其进一步提示，细胞之间的空间关系可能具有独立于细胞丰度的生物学和临床信息。

因此，空间组学带来的核心认识并非简单地为单细胞亚群“增加坐标”，而是将脑转移TME重新定义为由特定细胞状态、局部互作网络和代谢/基质条件共同组织形成的空间功能单元。目前这些生态位主要来自成熟脑转移灶的横断面分析，其形成顺序、稳定性以及治疗前后的动态变化仍需要纵向和功能性研究验证。

## 4 NSCLC脑转移TME与治疗反应及临床转化

NSCLC脑转移的治疗反应不仅取决于肿瘤细胞本身，也受到脑内血管、免疫及胶质细胞等TME因素影响，这些因素进一步构成治疗抵抗、联合干预及患者分层的重要依据。

### 4.1 脑转移TME与现有治疗反应

肺癌脑转移的治疗反应并不完全由药物进入中枢神经系统的能力决定，局部TME同样可影响残存肿瘤细胞的存活与耐药形成。既往研究提示，血管周生态位可促进EGFR突变脑转移细胞在奥希替尼治疗后的持续存活^[33]^，而小胶质细胞相关信号也可降低放疗敏感性^[42]^。这些结果说明，颅内耐药不仅是药物递送问题，也涉及脑内生态位对肿瘤细胞的持续支持。

免疫治疗同样受到局部免疫状态影响。脑转移灶中记忆T细胞减少、应激性T细胞及特定TAMs亚群富集等特征与较差的免疫检查点抑制剂（immune checkpoint inhibitors, ICIs）疗效有关^[43]^。临床研究^[54]^进一步显示，NSCLC脑转移患者接受程序性细胞死亡受体1（programmed cell death 1, PD-1）/PD-L1抑制剂单药的获益相对有限，而联合治疗可获得更好的生存结局。因此，脑转移治疗反应应理解为药物效应与局部TME共同选择的结果，但目前TME特征与具体治疗获益之间仍以相关性证据为主。

### 4.2 靶向脑转移TME的治疗策略

靶向脑转移TME的临床转化目前更适合作为现有治疗的联合策略，而非独立干预。研究^[55]^显示，酪氨酸激酶抑制剂（tyrosine kinase inhibitors, TKIs）治疗可诱导适应性免疫抑制，而细胞毒性T淋巴细胞相关蛋白4（cytotoxic T lymphocytes associated antigen 4, CTLA-4）阻断联合TKIs能够增强EGFR突变脑转移模型中的肿瘤控制；近期研究^[56]^进一步表明，PD-1与CTLA-4双重阻断可改善脑转移中的免疫排斥状态，并增强CD8^+ ^T细胞反应及三级淋巴结构形成。这些结果提示，针对治疗过程中形成的免疫抑制状态进行联合干预，可能是提高颅内疗效的重要方向。

血管TME同样具有潜在转化价值，抗血管生成治疗可能通过改善血管及免疫状态增强系统治疗效果，回顾性研究^[57]^也提示其与化疗和ICIs联合可能改善NSCLC脑转移患者生存。相比之下，针对小胶质细胞、星形胶质细胞及其通讯轴的策略目前仍主要处于临床前阶段。

### 4.3 脑转移TME标志物与精准治疗

NSCLC脑转移的患者分层可能需要超越单一PD-L1表达或免疫细胞密度，进一步整合细胞状态及空间组织特征。既往研究^[43,53]^已显示，特定T细胞、TAMs状态及其空间分布与疗效或预后有关联。近期单细胞与空间转录组研究^[58]^进一步识别出富集于肿瘤边界的免疫抑制性MP1程序，提示空间化转录特征可能成为新的分层指标。

不过，这些候选标志物目前多来自回顾性或小样本组学研究，尚缺乏标准化检测及前瞻性验证。未来更有潜力的策略可能是联合评估细胞状态、空间位置与邻域关系，以提高脑转移风险及治疗反应分层的准确性。

## 5 总结与展望

NSCLC脑转移是肿瘤细胞状态变化与脑内血管、胶质、免疫及基质共同塑造的动态过程。部分脑转移相关状态可能在原发灶中已经存在，并在播散、脑内定植和后续生长中进一步被选择或重塑。单细胞与空间组学则将这种异质性推进至细胞状态、互作网络和空间生态位层面，为理解脑内适应、免疫逃逸及治疗反应提供了新的整体框架。

目前研究仍以成熟脑转移灶的横断面、小样本分析为主，且治疗背景和检测平台差异较大。脑转移相关状态究竟来源于原发灶预存、转移选择还是脑TME诱导的可塑性变化仍缺乏纵向证据；基于转录推断的细胞通讯和空间关系也需进一步功能验证。

未来应整合配对原发灶与脑转移灶的纵向单细胞、空间组学及多模态数据，将研究由单一细胞或通路推进至“细胞状态-互作网络-空间生态位-治疗反应”的整体框架，并筛选可稳定检测和重复验证的TME标志物，以支持脑转移风险预测、疗效分层和联合治疗。
